# A case of skeletal tuberculosis and psoas abscess: disease activity evaluated using ^18^ F-fluorodeoxyglucose positron emission tomography-computed tomography

**DOI:** 10.1186/1471-2342-13-37

**Published:** 2013-11-14

**Authors:** Yoshifumi Kimizuka, Makoto Ishii, Koji Murakami, Kota Ishioka, Kazuma Yagi, Ken Ishii, Kota Watanabe, Kenzo Soejima, Tomoko Betsuyaku, Naoki Hasegawa

**Affiliations:** 1 Division of Pulmonary Medicine, School of Medicine, Keio University, 35 Shinanomachi Shinjuku-ku, Tokyo 160-8582, Japan; 2 Division of Nuclear Medicine, Department of Diagnostic Radiology, School of Medicine, Keio University, Tokyo, Japan; 3 Department of Orthopaedic Surgery, School of Medicine, Keio University, Tokyo, Japan; 4 Center for Infectious Diseases and Infection Control, School of Medicine, Keio University, Tokyo, Japan

**Keywords:** PET-CT, Psoas abscess, Gravitation abscess, Skeletal tuberculosis, Extrapulmonary tuberculosis

## Abstract

**Background:**

Psoas abscess complicating tuberculous spondylitis is a rare morbidity in extrapulmonary tuberculosis. There are no established guidelines for evaluating the clinical response of psoas abscess. Although several studies have shown that positron emission tomography-computed tomography with ^18^ F-fluorodeoxyglucose can play a potential role in diagnosing multifocal tuberculosis and monitoring the clinical response of pulmonary tuberculosis, to our knowledge, this is the first report demonstrating that positron emission tomography-computed tomography is useful for evaluating local inflammation and disease activity of a tuberculous psoas abscess.

**Case presentation:**

We report a case of multifocal bone and lymph node tuberculosis with concomitant lumbar psoas abscess in a 77-year-old man, along with a literature review. An initial positron emission tomography-computed tomography scan showed intense ^18^ F-fluorodeoxyglucose accumulation in the sternum, ribs, vertebrae, and lymph nodes. The patient was successfully treated with antitubercular agents and computed tomography-guided drainage therapy. A follow-up positron emission tomography-computed tomography after abscess drainage and 9 months of antitubercular drug treatment revealed that the majority of lesions improved; however, protracted inflammation surrounding the psoas abscess was still observed. These results indicate that disease activity of psoas abscess can remain, even after successful drainage and antitubercular medication regime of appropriate duration.

**Conclusion:**

We have successfully followed up the extent of skeletal tuberculosis complicated with psoas abscess by positron emission tomography-computed tomography. In this patient, positron emission tomography-computed tomography is useful for evaluating the disease activity of tuberculous psoas abscess and for assessing the appropriate duration of antitubercular drug therapy in psoas abscess.

## Background

Skeletal tuberculosis accounts for 10–20% of all cases of extrapulmonary tuberculosis, and almost 2% of all tuberculous infections [1,2]. Skeletal tuberculosis sometimes develops as multiple lesions and is more common in the elderly. The most common form of skeletal tuberculosis is tuberculous spondylitis (Pott’s disease), which comprises approximately half of all skeletal tuberculosis infections. Tuberculous spondylitis most commonly appears in the lumbar and thoracic spine, especially in the thoracolumbar region. Typically, it presents with low back pain that gradually increases over weeks, although the symptoms are sometimes latent and difficult to diagnosis. An accumulated abscess (abscessus frigidus) forms around the lesion, descending alongside the greater psoas muscle in the thoracolumbar or lumbar region, and it is referred to as a tuberculous psoas abscess [3]. The tubercular prevalence rate is on the decline, and psoas abscess has become a rare clinical picture, especially in developed and industrialized nations. There is no established evaluation protocol for monitoring the clinical response of skeletal tuberculosis to treatment [4].

Integrated functional modality in nuclear medicine has developed remarkably over the last few decades. Positron emission tomography-computed tomography (PET-CT) with ^18^ F-fluorodeoxyglucose (FDG) is regarded as a sensitive technique in oncological imaging. Several studies have shown a potential role for ^18^ F-FDG PET-CT in diagnosing multifocal tuberculosis and for monitoring the clinical response of pulmonary tuberculosis based on the characteristic uptake of ^18^ F-FDG by inflammatory and infectious lesions [5,6].

Here, we report a case of multifocal bone and lymph node tuberculosis with concomitant psoas abscess in an elderly patient.

## Case presentation

A 77-year-old male patient presented with a history of diabetes mellitus under oral diabetic agent treatment and was seen as an outpatient. He had symptoms of right axillary lymph node swelling and low back pain since 3 weeks. Except for the axillary adenopathy, findings on physical examination were unremarkable. Findings on contrast CT of the body showed generalized lymphadenopathy (bilateral supraclavicular, mediastinal, and abdominal paraaortic lymph nodes) and multiple bone lesions. The patient was afebrile, and other vital signs were unremarkable. Biopsy of the axillary lymph node was performed due to suspicion of malignant lymphoma. Granulomatous change and a marked necrosis were observed in the tissue. Although lymph node tuberculosis was suspected, *Mycobacterium* was not isolated from the tissue and there were no lesions in the lungs. Since he was of advanced age, the patient was taken under progressive observation without invasive study.

Two months later, he suffered higher brain dysfunction following head injury and was admitted to our hospital. Bone biopsy was carried out on a thoracic vertebral body with multiple bone lesions. Histopathology showed granulomatous inflammation in the medullary cavity. Based on histology of the lymph node and the bone lesion, he was diagnosed with bone and lymph node tuberculosis and was started on a combination treatment of isoniazid (300 mg/day), rifampicin (450 mg/day), and ethambutol (750 mg/day). Since he was of advanced age, he was not prescrived pyrazinamide.

**Figure 1:**
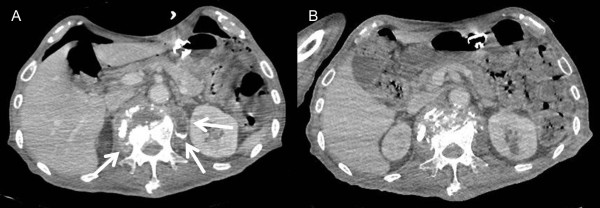
**Computed tomography (CT) findings of the multifocal bone and lymph node tuberculosis with concomitant psoas abscess.** Contrast CT images of a comminuted fracture of the L1 vertebral body and abscesses in the bilateral psoas major muscles (arrows) performed **(A)** before treatment and **(B)** after percutaneous abscess drainage and 6 months of antitubercular drug treatment.

One month after the start of medical treatment, findings on the contrast CT showed a comminuted fracture of the L1 vertebral body and abscess into the bilateral psoas major muscle at the level of L1 to L2 (Figure 1A). Furthermore, findings on contrast magnetic resonance imaging (MRI) showed that the psoas abscess was exerting pressure on the dural sac at L1/2. The left psoas was aspirated by percutaneous CT-guided drainage. About 15 mL of grayish white pus was drained. Gram staining showed multiple white blood cells, but no organisms. The smears were positive for acid-fast bacilli. Results of the tuberculosis polymerase chain reaction (TB-PCR) assay were positive. The culture eventually yielded *Mycobacterium tuberculosis* 2 months later, which was sensitive to all antituberculous medications except pyrazinamide. After drainage, the abscess cavity was reduced and the pressure against the dural sac resolved. After this intervention and 6 months of antimycobacterial therapy, contrast CT revealed no evidence of psoas abscess without bone fragment (Figure 1B).

**Figure 2:**
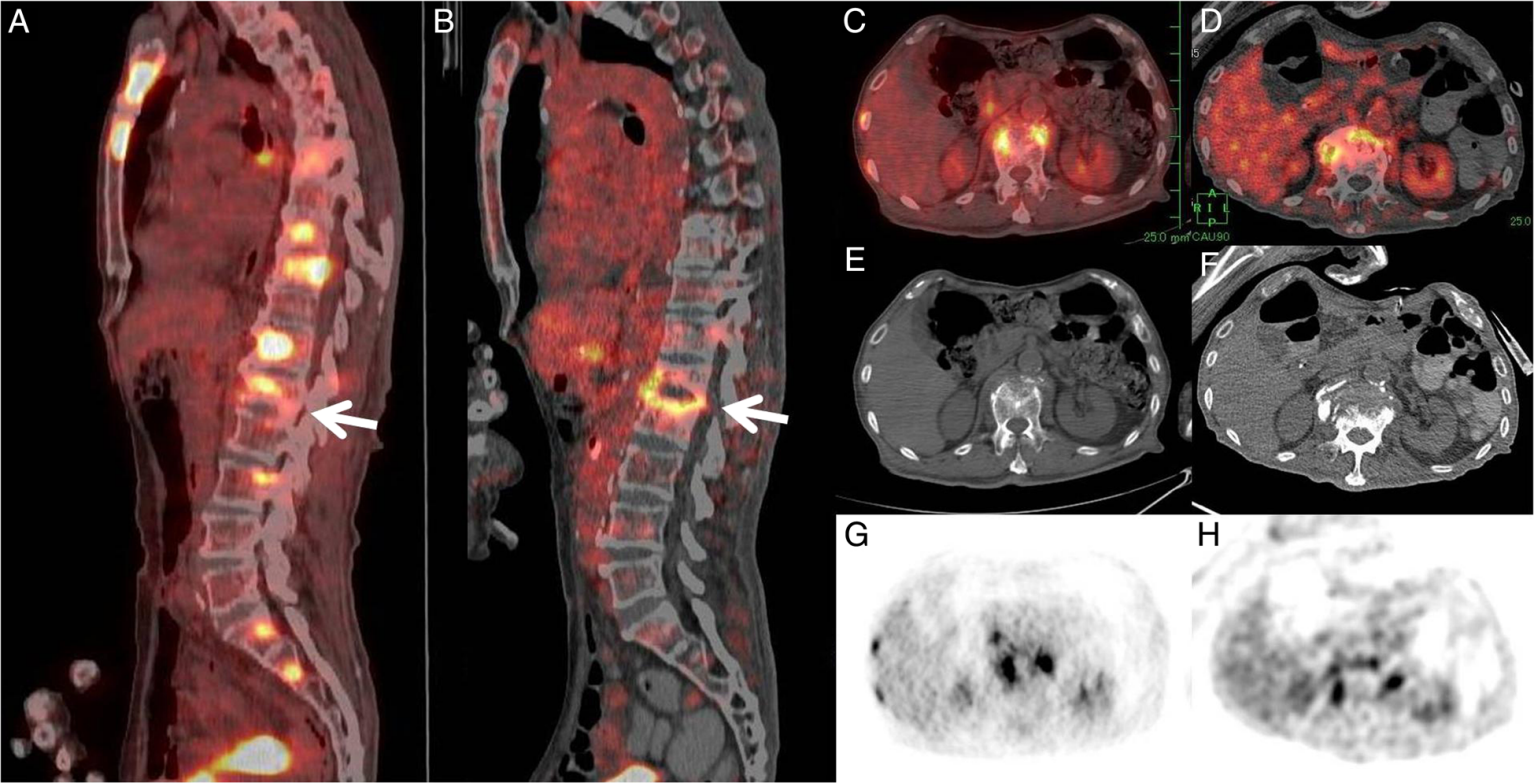
**Multifocal bone and lymph node tuberculosis with concomitant psoas abscess demonstrated on positron emission tomography-computed tomography (PET/CT).** The sagittal **(A, B)** and transverse **(C, D)** PET/CT fusion images, theaxial unenhanced CT images **(E, F)** and the FDG-PET images **(G, H)** showed persistent FDG uptake corresponding to the L1 vertebral body destruction (arrow) and abscesses in the bilateral psoas major muscles before **(A, C, E, G)** and after psoas abscess drainage and 9 months of antituberculous medications **(B, D, F, H)**.

To evaluate disease activity, PET-CT was performed and the images before treatment (Figure 2A, C) and after percutaneous abscess drainage and 9 months of antitubercular drug treatment were compared (Figure 2B, D). Before treatment, accumulation of FDG was observed in many lesions, including those in the sternum, ribs, vertebrae, and lymph nodes. These lesions improved after treatment; the average maximum standardized uptake value (SUV max) decreased from 18.8 (before treatment) to 2.1 (after drainage and 9 months of drug treatment) (11.1%). In these lesions, the highest accumulation was observed in the manubrium, and the SUV max decreased from 21.3 to 2.0 (9.4%). However, there was protracted accumulation of FDG in the region of vertebral body destruction and tuberculous psoas abscess formation (SUV max, from 15.8 to 4.82) (30.5%), which suggested incomplete resolution of the tuberculous inflammation (Figure 2B). We have also provided unenhanced CT (Figure 2E, F) and FDG-PET non-fusion images (Figure 2G, H) that confirmed hypermetabolism in the abscess.

## Discussion

Psoas abscess complicating tuberculous spondylitis is a rare morbidity in extrapulmonary tuberculosis. Various symptoms may be observed when it becomes severe, such as oppressive percussion tenderness of the spinous processes due to vertebral destruction, reflective spasm of the paraspinal muscles, and deformities of the vertebrae (kyphosis) and hip joints. Furthermore, the collapse of an abscess lump into the spinal canal or the sequestrum may cause neuropathy (Pott’s paralysis) due to pressure on the spinal roots. In the present case, the patient did not present with some typical symptoms; further, the symptoms were relatively mild. Hence, diagnosing the condition was difficult.

A definitive diagnosis of skeletal tuberculosis can be made based on findings of the culture and pathological tests of the infected tissues; but, these cultures are positive at a rate of 50–75% only, making bacteriologic confirmation of the disease very difficult [7]. Acid-fast staining is usually negative. Further, in this case, it was difficult to detect the tubercle bacilli until we performed percutaneous drainage of the psoas abscess. Moreover, we had to start the treatment on the provisional ground of pathological findings alone. Treatment of skeletal tuberculosis is based on antituberculous therapy applied to expectoration smear-positive tuberculosis. The optimal duration of therapy for treatment of skeletal tuberculosis is uncertain. For most patients, the antituberculous therapy has a sufficient effect similar to that for pulmonary tuberculosis and the recurrence is low [8]. However, a treatment duration of 9–12 months is particularly recommended for patients when the regimen does not contain rifampin and/or when they present with extensive or advanced disease [9,10]. A 12-month treatment course is recommended by the American Academy of Pediatrics [11]. These prolonged treatment periods are not based on a significantly high incidence of recurrence, but are determined in consideration of the seriousness of the recurrent case. In this case, we initiated a prolonged treatment phase for a patient with advanced disease, which we plan to continue for 12 months considering the results of PET-CT. The opinion on the necessity of surgical intervention for skeletal tuberculosis remains divided. Surgery is considered to be clinically indicated in cases involving high risk for neurological deficits and if severe kyphosis is observed in Pott’s disease [12]. Since the role of surgery in multifocal skeletal tuberculosis, as in this case, was not clear, we performed percutaneous CT-guided drainage and achieved a successful size reduction of the abscess cavity in combination with antitubercular agents.

There are no established guidelines for the evaluation of the clinical response to treatment of bone tuberculosis. Information on the role of clinical indicators and serological inflammatory markers in monitoring the clinical response are limited; neither is it useful to employ serial radiographic findings [4]. However, in a recent report, PET-CT was considered useful in the diagnosis and measurement of the treatment response of extrapulmonary tuberculosis [6]. In the current report, we successfully followed up the progress of skeletal tuberculosis complicated with psoas abscess by PET-CT before and after treatment. Although there are many reports of skeletal tuberculosis cases with psoas abscess, to our knowledge, this is the first report on the use of PET-CT for following disease activity. Although the usefulness of PET-CT may be limited, we observed the uptake of FDG surrounding the lesion, where the bone splinter remained after regression of the tuberculous psoas abscess. It is possible that this FDG accumulation represents local inflammation rather than an absolute proof of failure of the antitubercular treatment. However, considering that the principle of tuberculosis treatment is to prevent the development of drug resistance and relapse of disease, we decided that continuation of antitubercular treatment was appropriate. Regarding the treatment for tuberculous psoas abscess, we believe that disease activity can sometimes remain, even after successful drainage and antitubercular medication regime of appropriate duration. Therefore, we suggest that PET-CT may be useful in following this condition.

## Conclusions

We presented a case of skeletal tuberculosis complicated with psoas abscess which was successfully followed up by PET-CT. In this case, PET-CT is useful for evaluating the disease activity of tuberculous psoas abscess and for evaluating the appropriate duration of antitubercular drug therapy in psoas abscess.

## Consent

Written informed consent was obtained from the patient for publication of this Case report and any accompanying images. A copy of the written consent is available for review by this journal.

## Abbreviations

PET: Positron emission tomography; CT: Computed tomography; FDG: Fluorodeoxyglucose; MRI: Magnetic resonance imaging; SUV: Standardized uptake values.

## Competing interests

The authors declare that they have no competing interests.

## Authors’ contributions

YK drafted the manuscript. YK, MI, KIshioka, KY, KIshii, KW, KS, TB, and NH contributed to the diagnosis and treatment. KM reviewed the radiological findings and interpreted the data. YK, MI, KIshii, and KY contributed to the obtainment of the written informed consent. NH conceived the study. MI, TB, and NH reviewed the manuscript. All the authors approved the final version of the manuscript.

## Pre-publication history

The pre-publication history for this paper can be accessed here:

http://www.biomedcentral.com/1471-2342/13/37/prepub
